# Supplementary figures and images for: Constitutive activation of NF-κB inducing kinase (NIK) in the mesenchymal lineage using Osterix (Sp7)- or Fibroblast-specific protein 1 (S100a4)-Cre drives spontaneous soft tissue sarcoma
Source: PLoS One. 2021 Jul 22;16(7):e0254426. doi: 10.1371/journal.pone.0254426 (PMC8297882; doi:10.1371/journal.pone.0254426)

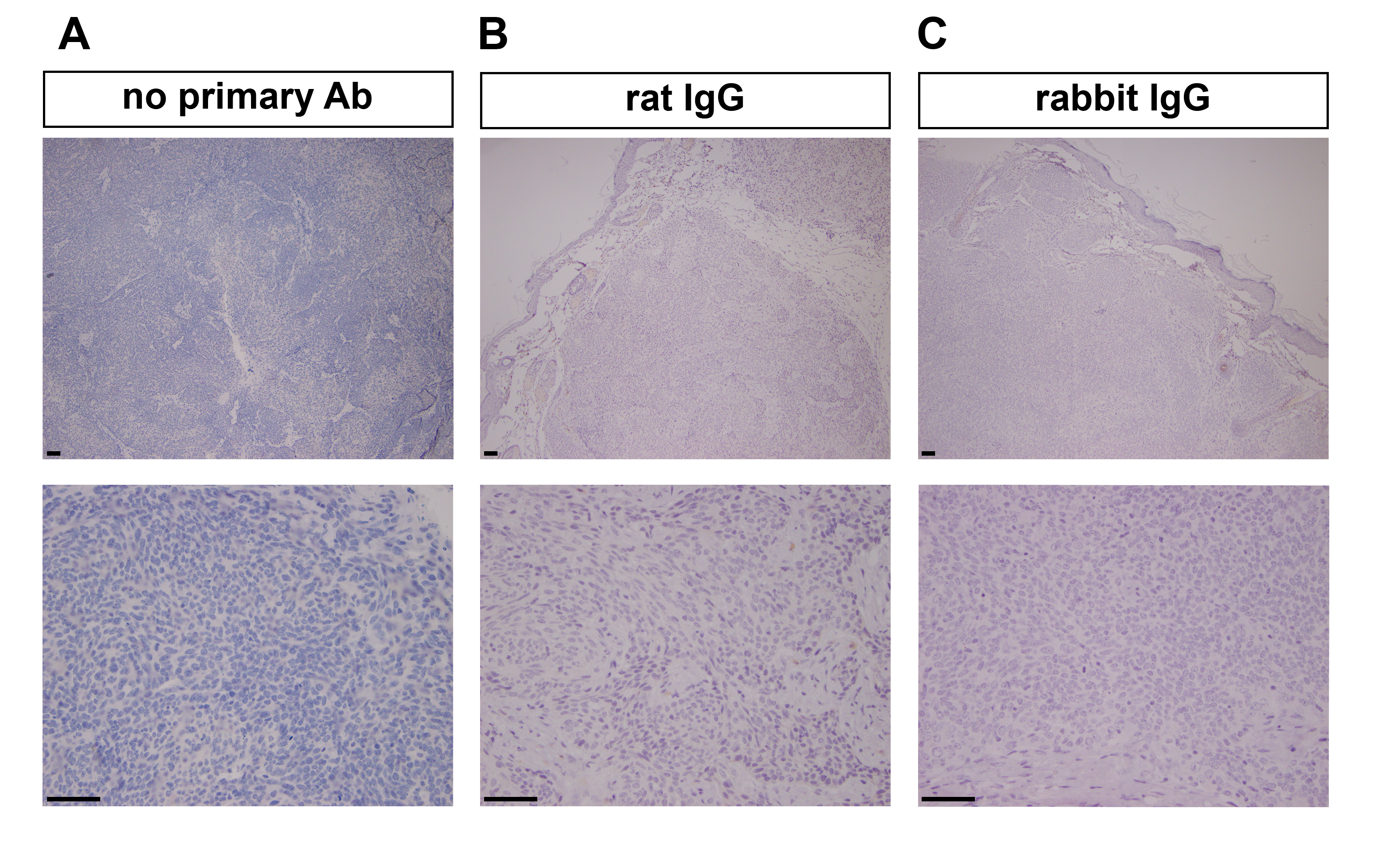

Supplement: S1 Fig — (A) No primary antibody stain for anti-goat GFP-biotin antibody. (B) Rat IgG background staining for CK19 antibody. (C) Rabbit IgG background staining for αSMA, vimentin, CD45, and S100 protein antibodies. All negative control stains were performed on Osx-Cre;NT3-tumor sections. Top row for each panel is 10x and the bottom row for each panel is 40x magnification. Scale bars = 100μM. (TIF) [file pone.0254426.s001.tif]

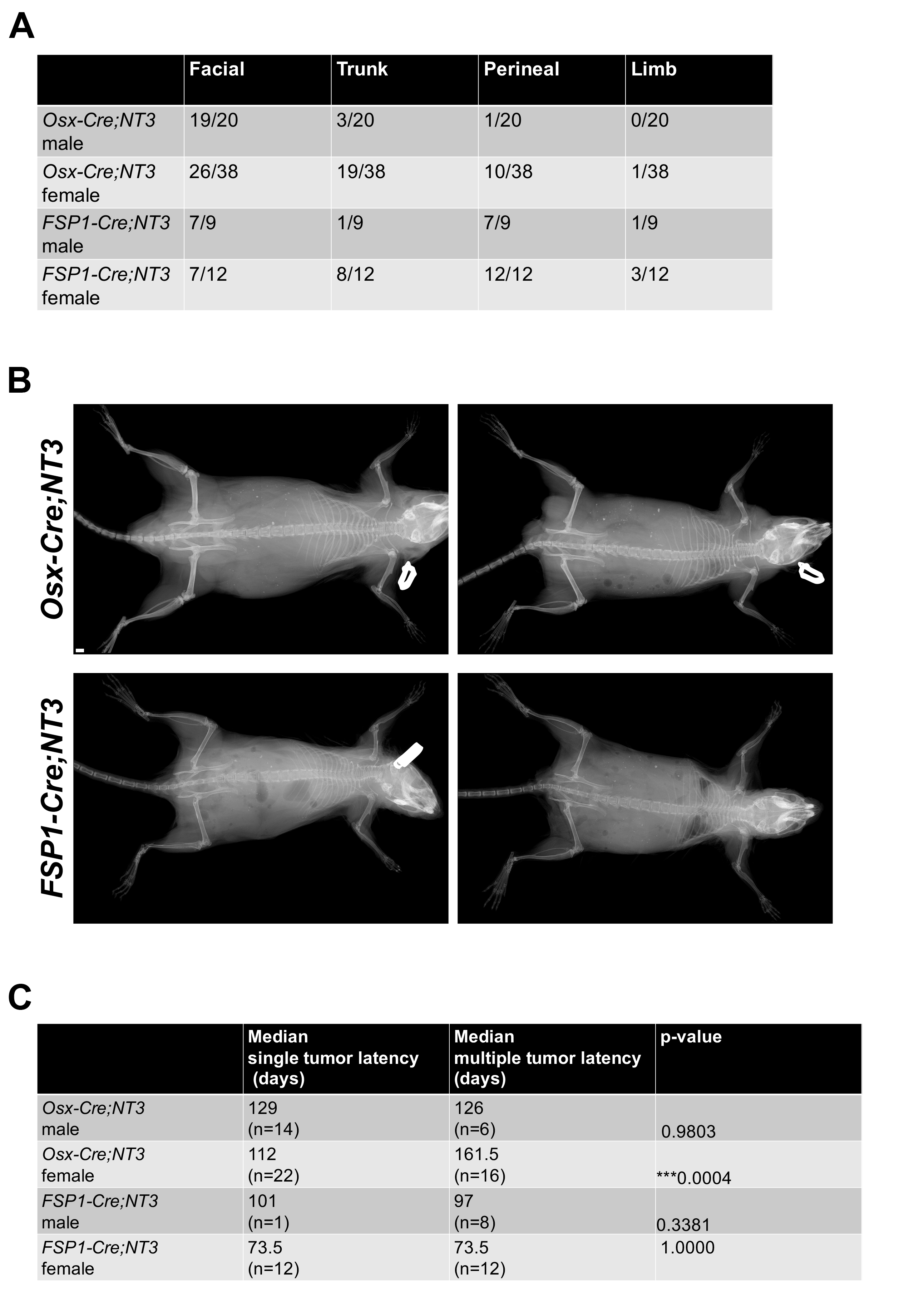

Supplement: S2 Fig — (A) Number of Osx-Cre;NT3 and FSP1-Cre;NT3 mice presenting with a tumor mass at different anatomical locations. In the case of multiple tumors within the same animal, the animal was counted once in each category for 1 or more masses at a given location. (B) Representative radiographic images revealing no overt osseous tumors in either strain. scale bar = 3mm. (C) Median tumor latency for Osx-Cre;NT3 and FSP1-Cre;NT3 animals presenting with single or multiple (≥2) tumors. Samples sizes are as indicated in each panel. Standard log-rank (Mantel-Cox) test: ****p<0.0001. (TIF) [file pone.0254426.s002.tif]

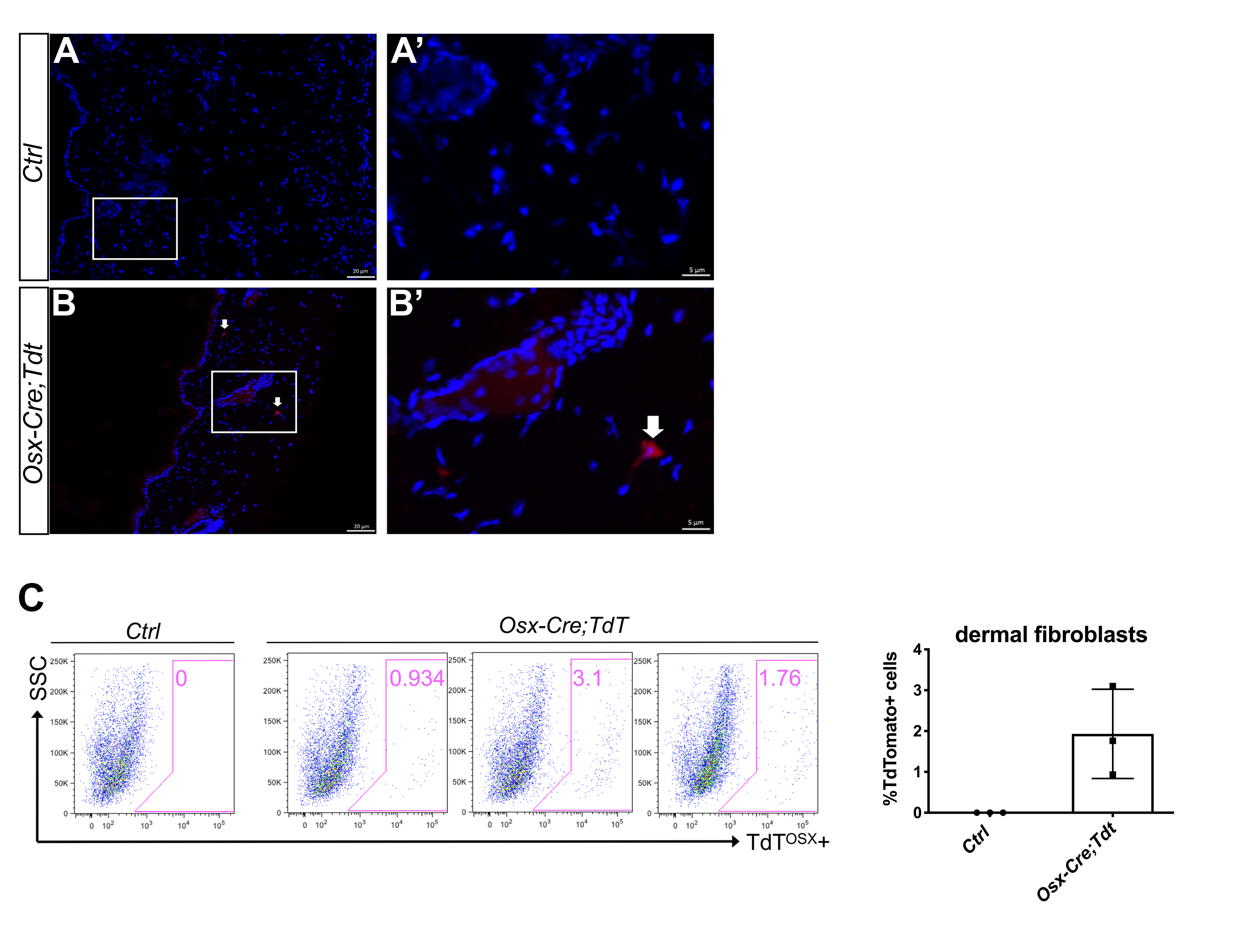

Supplement: S3 Fig — (A) Direct fluorescence of Tdt with DAPI counterstain in frozen skin sections from Ctrl or (B) Osx-Cre;Tdt mice. Representative 20x images shown with white arrows denoting Tdt+ cells. Scale bars = 20μm. (A’-B’) Higher magnification of boxed areas in A-B. Scale bars = 5μm. n = 2 each genotype. (C) Flow cytometry analysis for Tdt in cultured skin fibroblasts from Ctrl or Osx-Cre;Tdt mice. n = 3 each genotype. Unpaired one-tailed t-test with Welch’s correction: *p<0.05. (TIF) [file pone.0254426.s003.tif]

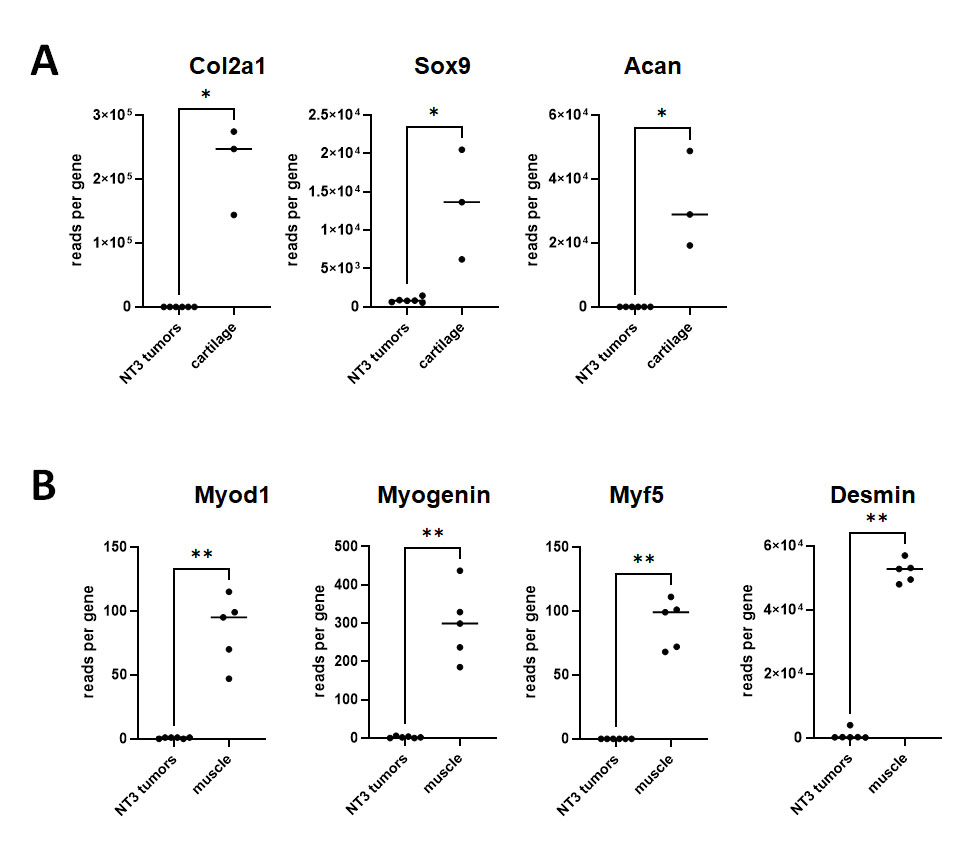

Supplement: S4 Fig — (A) Expression of cartilage markers or (B) muscle markers in NT3 tumors versus the respective tissue. Log10(reads per gene) is shown from normalized RNA-Seq data. Mann Whitney U test; *, p<0.05, **p<0.01. (JPG) [file pone.0254426.s004.jpg]

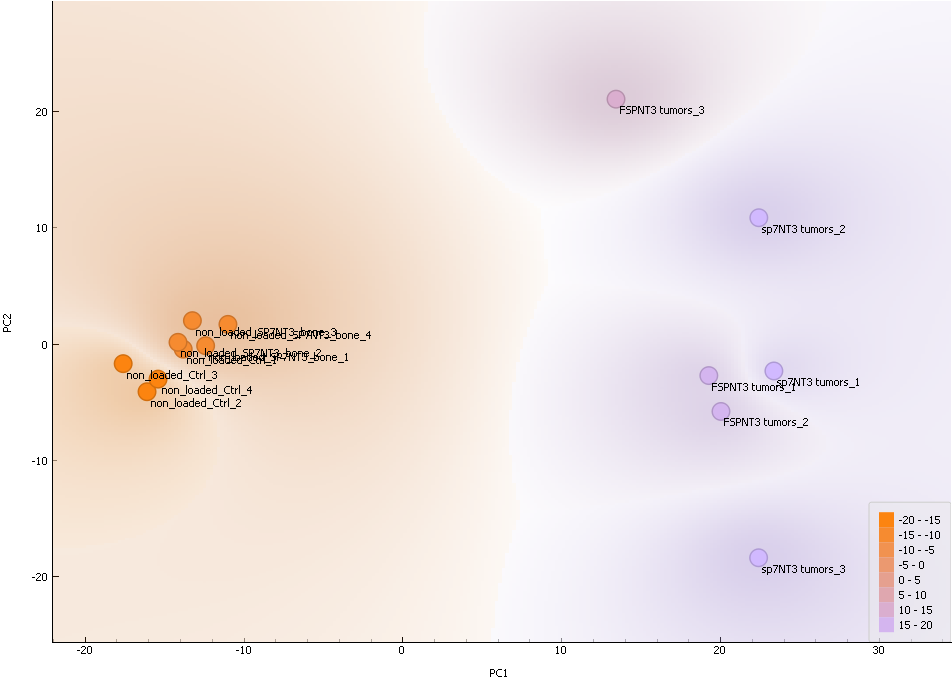

Supplement: S5 Fig — Principal component analysis of Osx-Cre;NT3 tumors, FSP1-Cre;NT3 tumors, Osx-Cre;NT3 bone, and Ctrl bone using genes from a gene set enrichment analysis (GSEA) of top NF-κB related gene sets listed in S8 Table. (PNG) [file pone.0254426.s005.png]

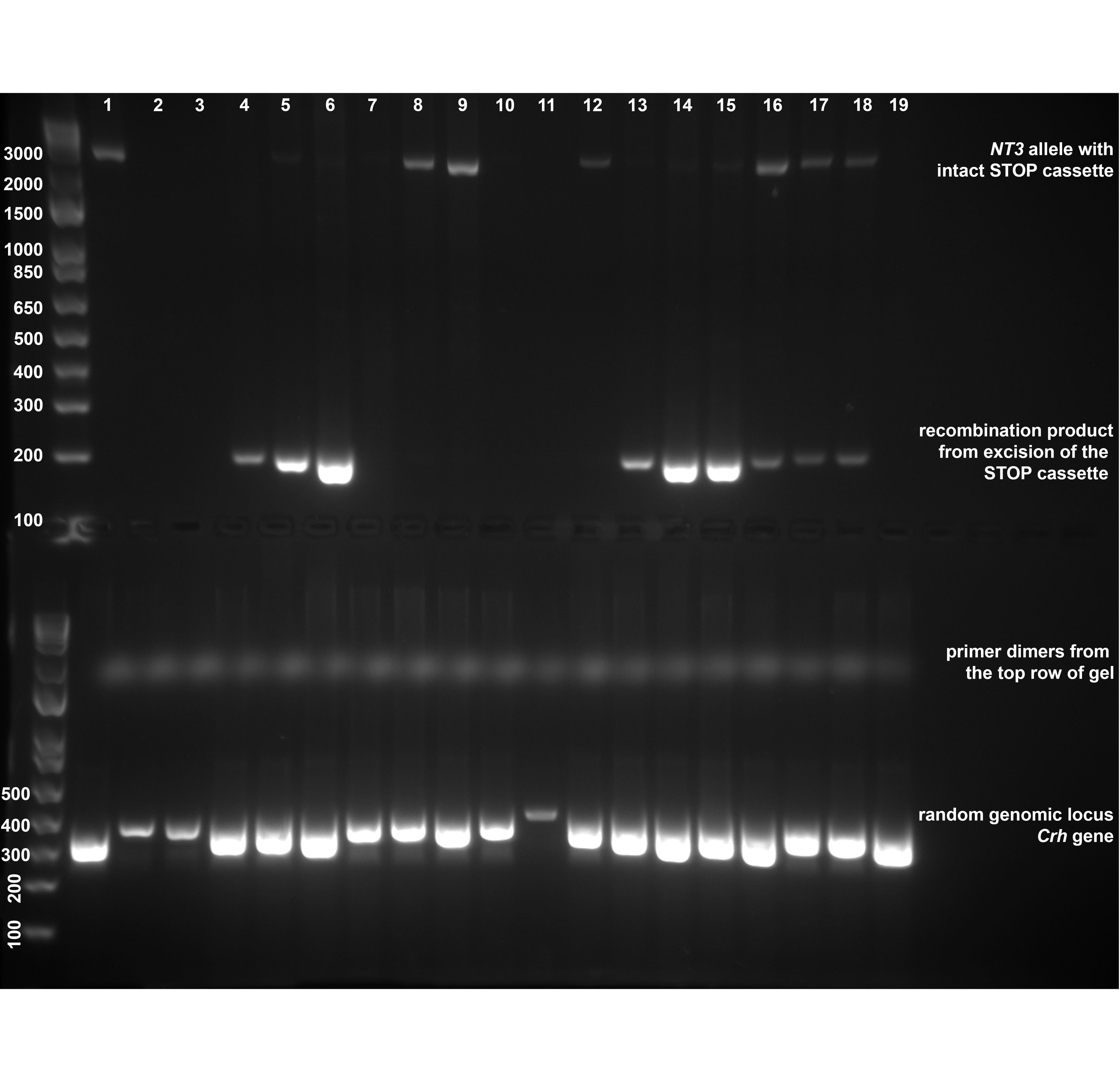

Supplement: S6 Fig — (TIF) [file pone.0254426.s006.tif]
