# Supplementary material for: Constitutive activation of NF-κB inducing kinase (NIK) in the mesenchymal lineage using Osterix (Sp7)- or Fibroblast-specific protein 1 (S100a4)-Cre drives spontaneous soft tissue sarcoma
Source: PLoS One. 2021 Jul 22;16(7):e0254426. doi: 10.1371/journal.pone.0254426 (PMC8297882; doi:10.1371/journal.pone.0254426)
Supplement: S1 File — (DOCX) [file pone.0254426.s015.docx]

**Supplementary Methods:**

*Radiographic imaging*

Whole body prone images were captured at 1.5x magnification using a Faxitron Ultrafocus 100 (Faxitron Bioptics, LLC, Tucson, AZ, USA). Dosage per animal: 45.0 kV, 0.24mA, 2.06 mAs, 8.42s, 0.64s, 0.00s. Sample size: *Osx-Cre;NT3* (n= 4 male; n=10 female) and *FSP1-Cre;NT3* (n=4 male; n=4 female). Images shown are of an *Osx-Cre;NT3* male, age 152 days (3 facial and 1 trunk tumor), *Osx-Cre;NT3* female, age 129 days (3 facial, 1 trunk, and 1 perineal tumor), *FSP1-Cre;NT3* female, age 101 days (1 perineal and 1 facial tumor), and *FSP1-Cre;NT3* female, age 88 days (2 perineal and 1 limb tumor).

*Quantitative real-time PCR (RT-qPCR)*

Tumor RNA was isolated using the Direct-zol RNA kit (R2072; Zymo Research, USA) and quantified by NanoDrop 2000 (ND-2000; Thermo Fischer Scientific, USA). RNA was reverse transcribed into cDNA using the SuperScript III First-Strand Synthesis System (Invitrogen, USA). Gene transcript levels were assessed by the 2^-ΔΔCt^ method and normalized to *Gapdh* (housekeeping gene). Gene-specific primers are listed in Supplementary Table 4.

*Fluorescence imaging*

Dorsal skin tissue was dissected and frozen immediately in optimal cutting temperature compound (OCT compound; Tissue-Tek, VWR, UK) at -80C°. Sectioning (longitudinal, 5μm thickness) was performed by the Musculoskeletal Research Center Histology and Morphometry core at Washington University. Frozen histological sections were brought to room temperature, rinsed in 1X PBS, and coverslipped with Prolong Diamond Antifade Mountant with DAPI (Thermo Fisher Scientific, P36962). Fluorescent images were obtained on the Zeiss Axio Imager.D2 microscope using a 20x objective with the DAPI and mCherry filters to visualize nuclear and Cre activated tdTomato expression, respectively. Ctrl and Osx-Cre;Tdt sections were imaged using the same exposure time and the same thresholds were applied to each image to maintain a black background and positive cell associated signal. n=2 each genotype, mixed male and female, age 12 weeks.

*Dermal fibroblast culture and flow cytometry*

Skin fibroblasts were isolated by removing the dorsal skin. The tissue was minced, then transferred to a conical tube containing collagenase solution (2mg/ml Collagenase A - Roche Diagnostics, 1mg/ml hyaluronidase - Sigma-Aldrich, 2U/ml DNase I - Sigma-Aldrich, in serum-free DMEM). The skin was digested on a rotisserie for 4 hours at 37°C. Following digestion, the cells were plated in a tissue culture coated plate and 24 hrs later the non-adherent cells were removed. Skin fibroblasts were expanded in culture for two weeks before flow cytometry. Adherent skin fibroblasts were trypsinized for 5 minutes at 37°C and washed and resuspended in FACS buffer (0.5% BSA, 2mM EDTA, 0.1% NaN3 in PBS). Samples were acquired using the BD LSR-Fortessa cytometer and analyzed with FlowJo version 9.3.2. n=3 each genotype, male, age 8 weeks. Unpaired one-tailed t-test with Welch’s correction.

*Gene Set Enrichment Analysis (GSEA):*

GSEA analysis was performed for 3 comparisons (*Osx-Cre;NT3 tumors*, *FSP1-Cre;NT3* tumors, or *Osx-Cre;NT3* and *FSP1-Cre;NT3 tumors* vs *Ctrl* bone) with GSEA v4.1.0 screening for all available gene-sets (msigdb.v7.4.symbols.gmt) focusing on NF-κB related gene-sets using standard settings. Gene sets were further filtered to only those with a normalized enrichment score of ≥ 1.5 and shared between at least 2 of the 3 comparisons. Duplicate genes across gene sets were removed before generation of a NF-kB gene-related PCA plot (Supplementary Figure 5 and Supplementary Table 8).
