# Supplementary material for: Constitutive activation of NF-κB inducing kinase (NIK) in the mesenchymal lineage using Osterix (Sp7)- or Fibroblast-specific protein 1 (S100a4)-Cre drives spontaneous soft tissue sarcoma
Source: PLoS One. 2021 Jul 22;16(7):e0254426. doi: 10.1371/journal.pone.0254426 (PMC8297882; doi:10.1371/journal.pone.0254426)
Supplement: Attachment — Submitted filename: NT3 PLOS ONE_revision response_final.docx [file pone.0254426.s016.docx]

We thank the reviewers for their thoughtful consideration of our manuscript. Below are their *specific comments* and our detailed responses to each one. We believe that consideration of these issues has improved the manuscript considerably.

Reviewer #1: Manuscript Number: PONE-D-20-37281
*Major Issues
1) Nuclear RelB suggests activation of the non-canonical NF-kB pathway. However, without evaluating RelB staining in tissues that do not express NT3 transgene, it is hard to conclude the extent to which constitutive NIK activation contributes to nuclear RelB. I understand based on the information provided by the authors that sarcoma can develop throughout the body but in some defined regions. The authors should perform RelB staining using tissues from these regions or cells positive for FSP1/Osx derived from control mice (mice with either NT3 alone or Cre alone) which do not express NT3 transgene. It is reassuring to see that stromal cells are negative for RelB in Figure 3 however, without knowing the identity of those cells it is hard to make a conclusion about nuclear RelB level in cells that do not express NT3 transgene. In addition, the authors should use at least another independent approach to confirm that the non-canonical pathway is active. For example, they could determine the processing of p100 to p52. Another suggestion is to perform a systematic analysis of expression of genes that are known to be regulated by NIK using their RNA seq to provide further evidence for high NIK activity in tumor cells.*

Response: Our point is not that normal tissues do not have cells that activate alternative NF-kB and therefore have nuclear RelB, but that the tumors have strong and very uniform activation. Furthermore, it was not possible to examine RelB staining in tumors from Ctrl mice (non NT3 transgene setting) since Ctrl mice never developed tumors up to 1 year of age. We do not have RelB IHC in normal (non-transgenic) skin sections. However, the skin overlying FSP1-Cre;NT3 tumor 187 shows rare RelB+ cells by IHC which may be DFs that express the transgene. We have revised the RelB IHC figure (now Fig 6) to show more of the surrounding tissues for comparison, and included all 6 tumors stained. Additionally, we now show a set of 12 NF-kB regulated genes that are more highly expressed in the tumors than in our panel of normal tissues as well as in the littermate control non-transgenic bone from our prior study, according to RNA-Seq data (now Fig 5). We consider this to be very strong evidence of NF-kB activation in the tumors, in conjunction with the high levels of nuclear RelB.

*The observation that RelB is nuclear does not exclude the possibility that the canonical NF-kB pathway is also active in NT3 genetic background. It is established that NIK can activate both canonical and alternative pathways (Staudt 2010). Thus, the possibility that both pathways may contribute to tumor development still exists. Evaluating the status of the canonical pathway activation would be helpful to address this question. The presence of nuclear RelB in tumor cells is not sufficient for an argument that tumor development is driven by the non-canonical pathway. To make such a claim, the authors should disrupt the activation of the non-canonical pathway and demonstrate reduced propensity for tumor formation or at least provide sufficient evidence that the non-canonical pathway is more active in tumor cells compared to healthy tissues along with the status for the canonical pathway. In the case where evidence is lacking, the authors should appropriately revise the arguments and include careful discussion of the canonical and non-canonical pathway contribution to tumorigenesis.*

Response: We do not claim that the canonical or classical pathway is not activated by the NT3 transgene, as in fact we have observed canonical events such as p65 nuclear translocation[1], [2]. We have carefully worded the manuscript, and indicate only NF-kB activation, not specifically alternative NF-kB, is driven by the NT3 transgene. Additional discussion of this point has been added, lines 607-615.

*2) The authors acknowledged that FSP1 promoter is not restricted only to fibroblasts. To address this concern, they stained tumor tissues with CD45 and concluded that CD45 staining is mostly from immune infiltrates. However, this experiment alone does not resolve the issue. Mesenchymal marker staining and CD45 staining data only demonstrate that most tumor cells are of mesenchymal origin. It does not provide evidence to support the idea that cancer arose from NT3 expressing mesenchymal cells. How do we know that tumor development is a result of NT3 expression in fibroblasts but not due to the expression of NT3 in cells with hematopoietic or other lineages positive for FSP1? At a minimum, the authors should preform immunofluorescent staining to demonstrate that majority of tumor tissue is GFP+ and aSMA/vimentin+ since NT3 transgene carries GFP. Even with these data, the authors should carefully discuss the limitations of the data and possible alternative explanations for observed phenotypes.*

Response: We have shown that the majority of tumor cells are GFP+ (Fig 2) and aSMA+ (Fig 3), and we have indicated in the text the extent of staining for each. Our matched images of aSMA and CD45 from adjacent sections (Fig 3) show little overlap in these populations. Based on these findings, we find it extremely unlikely that the tumors could arise from NT3+/CD45+ cells. This is discussed on lines 585-587 in the discussion.

*3) The authors mentioned that they did not have a true control that they could compare to for the RNA seq data. Although I recognize the difficulty, they could have used non-cancerous cells positive for Osx or FSP1 sorted from mice or cell lines that are known to be active for these promoters. Ideally, this type of control should be used. Without a true control, analysis of the gene expression data is not robust enough to derive a confident conclusion. I appreciate their discussion on the limitations of their gene expression data.*

Response: We have directly compared the tumors to Osx-Cre;NT3 bones, and they show a similar, but more dramatic, increase in expression of NF-kB target genes in the new Fig 5B/C. Given the relative rarity of Cre+ cells in benign skin, it is not practical to isolate these cells to perform RNA-Seq.

*On a related note, it is unclear how fold changes were determined for differentially expressed genes. The authors stated “we compared both Osx-Cre;NT3 and FSP1-Cre;NT3 tumors together (NT3 tumors) vs all benign tissues shown in Fig 5A to generate a list of differentially expressed genes (DEGs).” However, in the excel file that is labeled as Suppl Table 6, formulae to calculate the fold changes were not provided. For complete transparency, the authors should provide this information as part of the excel sheet and detailed explanation of how the values were derived in the method section. For example, how were “mean normal counts” determined?*

Response: We have added more detailed methodology describing our calculations to the methods section (lines 238-242) as well as added a separate tab to Supplementary Table 6.

*4) IHC data: Quantification of the staining would be helpful to inform the reader of the variability and reproducibility of the staining. For instance, are most images 80% positive for the staining? What percentage of the tumors resembles the representative images? Quantification may be in the form of scoring of each image (low staining, medium staining or high staining) or percentage of positive cells per field (if that is feasible).*

Response: Additional description of the extent of staining has been added to the text describing IHC data in Figs 2, 3, and 6.

*5) It is puzzling and disconcerting to observe vimentin staining mostly in the periphery of FSP1-Cre; NT3 tumors unlike the staining for a-SMA. This raises the question of whether cells with active Osx or FSP1-promoter are positive for vimentin and a-SMA. The authors should address this question using Tdt mice that they used in their study with appropriate promoters driving Cre (to examine Tdt and vimentin/a-SMA positive cells). This can help provide some answers to the question regarding the lineage of the tumor.*

Response: We acknowledge that there are limitations to the IHC data. We previously showed that isolated tdT+ cells from Osx-Cre reporter mice also express a-SMA and Vimentin mRNA by q-RT-PCR [3]. We do not have mice with Tdt and the NT3 transgene so we cannot stain for these markers as well as Tdt, and also do not have FSP1-Cre;Tdt reporter mice available. It would take 5-6 months to generate such mice and wait for tumors to occur. However, our IHC for a-SMA and GFP on adjacent sections (from different tumors to those shown in Fig 3 clearly demonstrates a consistent pattern of overlapping staining for a-SMA and GFP (Reviewer Figure 1). We do not know why the vimentin is less uniformly expressed in the tumor cells from the FSP1-Cre line. Nevertheless, in conjunction with the fact that a diagnosis of poorly differentiated sarcoma is often one of exclusion, the totality of the evidence points towards a mesenchymal origin without differentiation towards a specific lineage.

**Reviewer Figure 1: Adjacent sections of tumors (different mice than in Fig 3), stained for GFP and αSMA.**

*6) In PCA, why did Osx-Cre; NT3 bone samples group away from Osx-Cre; NT3 tumor samples? Given that the protein product of NT3 transgene is responsible for NF-kB pathway activation, they should be at least similar in some aspects. This observation suggests that considerable changes not directly influenced by NT3 transgene occurred in tumors. Can the authors provide better insights into this?*

Response: The original PCA we showed, now Fig 4A, included all genes. In this analysis, specific pathway activation was not apparent while tissue specific expression patterns were, explaining why tumors segregated away from NT3+bone. In fact, even when we performed a gene set enrichment analysis (GSEA) of top NF-kB-related genes (approx. 500 genes) comparing only tumors and bone, tissue type seemed dominant to the transgene (new S5 Fig and new Supp Table 8). To further refine our analysis to only NT3 transgene target genes, we analyzed 12 genes found to be differentially expressed in age/sex-matched NT3 vs Ctrl bones that contained 2 or more κB response elements[2]. We found a similar pattern of upregulation between NT3 tumors and the normal tissues in our RNA-Seq panel (New Fig 5B-C), which we consider to be strong evidence of NT3 transgene activation as they were consistent with both Cre-drivers in the tumors.

*7) Due to the spontaneous nature, transgenic mouse models reported in this paper are not entirely genetically tractable. The authors recognized this aspect as well. Further characterization of genetic changes will be helpful to provide the relevance of this mouse model to human soft tissue sarcoma. This will also increase the utility of these mouse models in the field. For instance, the authors should characterize the status of some common genetic changes associated with soft tissue sarcoma (Dodd et al., 2010). The authors did make a statement that they did not observe notable changes in expression of genes whose alterations are associated with sarcoma. However, as the authors described, their differential gene expression data are limited by the lack of true controls. Thus, instead of relying on the RNA seq data, assessing the status of a few defined sarcoma-associated genetic changes would be helpful (e.g. Western blot analysis using tumor tissues and healthy tissues or cells positive for FSP1 or Osx)*

Response: The need for only a single transgene and 100% penetrance in a relatively short time window (tumors occur mainly between 3 and 5 months of age) makes this model easy to combine with other genes or pharmacological agents. Clearly, there is much more work that can be done to understand the nature of the tumors in our model, and the factors involved in their initiation. However, we consider this work beyond the scope of this initial report.

*Reviewer #2: The submitted manuscript by Davis et al presents two new models of mesenchymal tumors. These are exciting and potentially useful new models of sarcoma. I have two recommendations: 1) the authors should include a board certified pathologist or a veterinary pathologist (preferred) for a detailed assessment of IHC staining. If they have already done so it is not clear from the methods.*

Response: The senior author is a board certified pathologist with ~30 years of experience in the analysis of mouse and human tissues, now noted in methods on line 204.

*2) The gene ontology analysis appears to compare the NT3 tumors (though its not clear whether these are Osx or FSP driven tumors) to ALL the normal tissues in the PCA. Both the Fsp and Osx driven tumors have the closest relationship with connective tissue, which is perfectly reasonable. I think it might be more accurate to compare the tumors to the cartilage specifically and show that data.*

Response: We have clarified the grouping of NT3 tumors in the methods on lines 247-248. Although the PCA showed the NT3 tumors to be closest to cartilage, there is neither an anatomic connection of the tumors to any cartilaginous tissue nor any histologic features of cartilaginous differentiation, and expression of cartilage marker genes is very low (new S4 Fig). Therefore, we did not perform GO analysis between tumors and cartilage.

*In general. this short report of new sarcoma models taking advantage of deregulated non-canonical NF-kB signaling has the potential to be very useful to the field.*

Response: Thank you for your consideration of the value of this model.

*Reviewer #3: This manuscript by Davis et al. provides a descriptive account of a novel mouse model of soft tissue sarcoma driven by Osterix or Fibroblast-Specific Protein 1 lineage cells with constitutive activation of the NF-kB pathway. The sarcoma subtype does not have a clear human counterpart, which is a limitation but not surprising in the context of a genetic engineered mouse model. The work provides a novel mouse model of sarcoma and an opportunity to expand our understanding of the NF-kB pathway in sarcomagenesis. The study is well written and I do not have major concerns in its present form. I do have the following minor suggestions/questions:

1) In the description of where tumors arise in the mice, there is a range of for each location. Please clarify what this range means as I think it would be fine to just state the sample size and the observation.*

Response: The percentage range listed refers to the incidence in males vs. females, now clarified in the text.

*2) While the model system is highly penetrant, there is a comment made the rarity of tumors overall. Was there any thought to do a genomic analysis to assess for gene deletion/amplification? Any possible role for an environmental stimulus such as tissue injury? Is expression of the Osx or FSP higher in the face vs. other tissue types?*

Response: Although interesting, we consider analysis for gene amplification/deletion beyond the scope of the current study. We also have not directly compared Osx or FSP expression in skin on the face vs other sites. Injury and resulting inflammation, from grooming or fighting, may indeed play a role in tumorigenesis, by inducing expression of tumorigenic cofactors or enhancing NT3 transgene expression. We found BMP7 to be highly expressed in tumors, and this factor can be increased by inflammation in skin and induce Osx [4],[5]. However, in the course of our previous study on the Osx-Cre;NT3 mice we performed OVX surgeries and followed mice for 4 weeks, but did not observe any tumors at the surgical sites. Therefore, significantly more work needs to be done to resolve this issue.

*3) Was there any expression of myogenic markers in the tumors, ie myogenin or MyoD?*

Response: In the RNA-Seq analysis, expression of MyoD1, Myogenin, Myf5, and Desmin were all very low compared to the skeletal muscle samples, now shown in S4 Fig.

**References:**

[1]	C. Yang, K. McCoy, J. L. Davis, M. Schmidt-Supprian, Y. Sasaki, R. Faccio, and D. V. Novack, “NIK stabilization in osteoclasts results in osteoporosis and enhanced inflammatory osteolysis.,” *PLoS ONE*, vol. 5, no. 11, p. e15383, 2010.

[2]	J. L. Davis, L. Cox, C. Shao, C. Lyu, S. Liu, R. Aurora, and D. J. Veis, “Conditional Activation of NF‐κB Inducing Kinase (NIK) in the Osteolineage Enhances Both Basal and Loading‐Induced Bone Formation,” *Journal of Bone and Mineral Research*, vol. 34, no. 11, pp. 2087–2100, Aug. 2019.

[3]	B. Ricci, E. Tycksen, H. Celik, J. I. Belle, F. Fontana, R. Civitelli, and R. Faccio, “Osterix-Cre marks distinct subsets of CD45- and CD45+ stromal populations in extra-skeletal tumors with pro-tumorigenic characteristics,” *Elife*, vol. 9, pp. 7556–29, Aug. 2020.

[4]	I. Borek, R. Köffel, J. Feichtinger, M. Spies, E. Glitzner-Zeis, M. Hochgerner, T. Sconocchia, C. Krump, C. Tam-Amersdorfer, C. Passegger, T. Benezeder, J. Tittes, A. Redl, C. Painsi, G. G. Thallinger, P. Wolf, G. Stary, M. Sibilia, and H. Strobl, “BMP7 aberrantly induced in the psoriatic epidermis instructs inflammation-associated Langerhans cells.,” *J Allergy Clin Immunol*, vol. 145, no. 4, pp. 1194–1207.e11, Apr. 2020.

[5]	T. Sconocchia, M. Hochgerner, E. Schwarzenberger, C. Tam-Amersdorfer, I. Borek, T. Benezeder, T. Bauer, V. Zyulina, C. Painsi, C. Passegger, P. Wolf, M. Sibilia, and H. Strobl, “Bone morphogenetic protein signaling regulates skin inflammation via modulating dendritic cell function.,” *J Allergy Clin Immunol*, Oct. 2020.
