# Supplementary material for: Constitutive activation of NF-κB inducing kinase (NIK) in the mesenchymal lineage using Osterix (Sp7)- or Fibroblast-specific protein 1 (S100a4)-Cre drives spontaneous soft tissue sarcoma
Source: PLoS One. 2021 Jul 22;16(7):e0254426. doi: 10.1371/journal.pone.0254426 (PMC8297882; doi:10.1371/journal.pone.0254426)
Supplement: Attachment — Submitted filename: NT3 PLOS ONE_revision response_June.docx [file pone.0254426.s017.docx]

We thank the reviewers and editors of their careful consideration of our revisions. We have addressed each of the remaining issues raised by Reviewer 1, as explained below. For clarity, we have copied the previous comments and our original responses (labeled #1), as well as our new responses (labeled #2), and have omitted those considered resolved on the last round of review.

Reviewer #1: Responses to the authors
Manuscript Number: PONE-D-20-37281
Major Issues
1) Critique #1: Nuclear RelB suggests activation of the non-canonical NF-kB pathway. However, without evaluating RelB staining in tissues that do not express NT3 transgene, it is hard to conclude the extent to which constitutive NIK activation contributes to nuclear RelB. I understand based on the information provided by the authors that sarcoma can develop throughout the body but in some defined regions. The authors should perform RelB staining using tissues from these regions or cells positive for FSP1/Osx derived from control mice (mice with either NT3 alone or Cre alone) which do not express NT3 transgene. It is reassuring to see that stromal cells are negative for RelB in Figure 3 however, without knowing the identity of those cells it is hard to make a conclusion about nuclear RelB level in cells that do not express NT3 transgene. In addition, the authors should use at least another independent approach to confirm that the non-canonical pathway is active. For example, they could determine the processing of p100 to p52. Another suggestion is to perform a systematic analysis of expression of genes that are known to be regulated by NIK using their RNA seq to provide further evidence for high NIK activity in tumor cells.

Response #1: Our point is not that normal tissues do not have cells that activate alternative NF-kB and therefore have nuclear RelB, but that the tumors have strong and very uniform activation. Furthermore, it was not possible to examine RelB staining in tumors from Ctrl mice (non NT3 transgene setting) since Ctrl mice never developed tumors up to 1 year of age. We do not have RelB IHC in normal (non-transgenic) skin sections. However, the skin overlying FSP1-Cre;NT3 tumor 187 shows rare RelB+ cells by IHC which may be DFs that express the transgene. We have revised the RelB IHC figure (now Fig 6) to show more of the surrounding tissues for comparison, and included all 6 tumors stained. Additionally, we now show a set of 12 NF-kB regulated genes that are more highly expressed in the tumors than in our panel of normal tissues as well as in the littermate control non-transgenic bone from our prior study, according to RNA-Seq data (now Fig 5). We consider this to be very strong evidence of NF-kB activation in the tumors, in conjunction with the high levels of nuclear RelB.

Critique #2: The reviewer did not suggest tumors from Ctrl mice to be stained with RelB. The suggestion was to perform “RelB staining using tissues from these regions or cells positive for FSP1/Osx derived from control mice (mice with either NT3 alone or Cre alone) which do not express NT3 transgene”. The reviewer understands that mice with NT3 alone or Cre are simply controls in which NIK is not constitutively active and that transgene-driven tumors do not develop in these mice. Therefore, the reviewer understands that the isolation of healthy tissues from these mice does not require any waiting time and that these control mice are already in existence. Hence, the argument about tumor development taking up to 1 year of age confuses the reviewer.
Including the surrounding tissues help to ensure that RelB staining is specific to tumors. Independent assessment of NF-kB regulated gene expression in tumor tissues compared to the tissues without the transgene also supports the argument that NF-kB pathway is activated.

Response #2: We apologize for any misinterpretation of the reviewer’s previous comment. The intent of suggesting the aging of Cre-negative littermates to 1 year was to rule out a spontaneous mutation in our colony outside of the NT3 locus and independent of Cre-mediated effects. However, we minimized founder effects by using multiple breeding pairs and routinely backcrossing both NT3 and Osx-Cre lines to C57Bl/6 mice from JAX. Additionally, the tumor penetrance is 100%, so another driver mutation is highly unlikely.

We do not believe that performing IHC for RelB on non-transgenic skin would be a fruitful endeavor. Our images of NT3-transgenic skin near tumors and of the Osx-Cre reporter mice show very few positive cells in the dermis. A previous report by Hinata et al (ref 57, Fig 1) showed only rare cells in the dermis with NF-kB reporter activation. Therefore, in non-transgenic skin, background RelB expression, if present, would likely be even more rare than in tumor-adjacent NT3-transgenic tissue. Without co-staining for CD45 and fibroblast markers (at a minimum), it would be impossible to identify rare RelB-positive dermal cells, and thus, understand their relevance to tumorigenesis.

2) Critique #1: The authors mentioned that they did not have a true control that they could compare to for the RNA seq data. Although I recognize the difficulty, they could have used non-cancerous cells positive for Osx or FSP1 sorted from mice or cell lines that are known to be active for these promoters. Ideally, this type of control should be used. Without a true control, analysis of the gene expression data is not robust enough to derive a confident conclusion. I appreciate their discussion on the limitations of their gene expression data.

Response#1: We have directly compared the tumors to Osx-Cre;NT3 bones, and they show a similar, but more dramatic, increase in expression of NF-kB target genes in the new Fig 5B/C. Given the relative rarity of Cre+ cells in benign skin, it is not practical to isolate these cells to perform RNA-Seq.

Critique #2: The suggestion was to isolate non-cancerous cells positive for Osx or FSP1. For example, the authors reported that FSP-1 “predominantly shows expression in the fibroblast population of multiple organs but has also been documented in the myeloid lineage[31], [32], [43]-[45].” While it is understandable that the authors may not want to perform RNA seq experiments due to the time constraint, their response on “the relative rarity of Cre+ cells in benign skin” confuses the reviewer.

Response #2: It is not clear to us which population of Osx- or FSP1-positive cells this reviewer suggests for analysis. To us, the most relevant would be cells from benign skin, which are relatively rare in the reporter mice. We believe we have included sufficient data, presented with clear indications about its limitations, to warrant publication without further experimentation.


3) Critique #1: IHC data: Quantification of the staining would be helpful to inform the reader of the variability and reproducibility of the staining. For instance, are most images 80% positive for the staining? What percentage of the tumors resembles the representative images? Quantification may be in the form of scoring of each image (low staining, medium staining or high staining) or percentage of positive cells per field (if that is feasible).

Response #1: Additional description of the extent of staining has been added to the text describing IHC data in Figs 2, 3, and 6.

Critique #2: The descriptions added for those figures do not address the issue of reproducibility and variability. They simply describe what the reader can see in the images presented.

Response 2: We used ranges of positivity in the text along with indications of the strength (or range thereof), indicative of the variability we saw across the 6 separate tumors studied, now explicitly stated as (n=3 of each) in the text (line 410). All of the immunostaining was performed at the same time, so that it would be comparable across samples. If the journal allows a large file format, we would be happy to add scanned whole slide images for all of the IHC as supplemental information. Otherwise, we would certainly provide access to the images upon request. Unfortunately, there are no public databases in which to deposit staining data.

3) Critique #1: It is puzzling and disconcerting to observe vimentin staining mostly in the periphery of FSP1-Cre; NT3 tumors unlike the staining for a-SMA. This raises the question of whether cells with active Osx or FSP1-promoter are positive for vimentin and a-SMA. The authors should address this question using Tdt mice that they used in their study with appropriate promoters driving Cre (to examine Tdt and vimentin/a-SMA positive cells). This can help provide some answers to the question regarding the lineage of the tumor.

Response #1: We acknowledge that there are limitations to the IHC data. We previously showed that isolated tdT+ cells from Osx-Cre reporter mice also express a-SMA and Vimentin mRNA by q-RT-PCR [3]. We do not have mice with Tdt and the NT3 transgene so we cannot stain for these markers as well as Tdt, and also do not have FSP1-Cre;Tdt reporter mice available. It would take 5-6 months to generate such mice and wait for tumors to occur. However, our IHC for a-SMA and GFP on adjacent sections (from different tumors to those shown in Fig 3) clearly demonstrates a consistent pattern of overlapping staining for a-SMA and GFP (Reviewer Figure 1). We do not know why the vimentin is less uniformly expressed in the tumor cells from the FSP1-Cre line. Nevertheless, in conjunction with the fact that a diagnosis of poorly differentiated sarcoma is often one of exclusion, the totality of the evidence points towards a mesenchymal origin without differentiation towards a specific lineage.

Critique #2: The reviewer appreciates the explanation and the figure provided. The suggestion is to describe the limitation of vimentin staining under Discussion as well (line 572-573).

Response #2: Discussion has been amended to note variability of vimentin staining (lines 572-575).
